# Sociodemographic determinants of child mortality based on mothers’ attitudes toward partner violence: Evidence from Bangladesh

**DOI:** 10.1016/j.heliyon.2023.e13848

**Published:** 2023-02-25

**Authors:** Ferdous Ara, Mir Misnad Sultana, Sabrina Naoshin, Israth Sultana, Mohammad Nazmul Hoq, Mohammad Emdad Hossain

**Affiliations:** a Department of Computer Science and Engineering, BGC Trust University Bangladesh, Chandanaish, Chattogram, Bangladesh; b Department of Business Administration, BGC Trust University Bangladesh, Chandanaish, Chattogram, Bangladesh; c Instructor of Mathematics, Pre-UG, Asian University for Women, Chattogram, Bangladesh; d Department of Business Administration, International Islamic University Chittagong, Bangladesh

**Keywords:** Child mortality, Demographic and socioeconomic factors, Partner violence, Bangladesh

## Abstract

Bangladesh, a lower-middle-income country in South Asia, has achieved a significant reduction in child mortality over the last three decades from 151 to 40 per 1000 live births. However, child mortality is still considered high, which may be attributed to a lack of awareness among mothers regarding the risk factors, particularly their perceptions of intimate partner violence (IPV). To investigate the effect of demographic and socioeconomic factors of women on child mortality, this study extracted data from the cross-sectional survey of Bangladesh Multiple Indicator Cluster Survey (MICS) 2019. The data were analyzed using a Poisson regression model to assess women’s perceptions and exposure to physical violence in the last three years preceding the survey and their impact on the risk of child mortality. The results indicated that approximately 26% of the participants justified domestic violence under certain circumstances. Moreover, the prevalence of child mortality was higher among those who thought that IPV is acceptable than among those who considered such violence to be unjustified. Among women who are strongly averse to partner violence, the risk of child mortality was significantly lower in those who had higher levels of education, higher household income, internet usage experience, first child at 20 years or later, frequent access to mass media, and one or two children ever born. Moreover, child mortality rates also varied across geographical areas, with children from Sylhet and Mymenshing being the most vulnerable. The data indicates women’s intolerable attitudes toward partner violence not only improve their status but also increase the survival chances of their young children.

## 1 Introduction

Child mortality (death of a child under 5 years) is a significant determinant of development in a society, and a country's principles and interests are also reflected in how it handles infant mortality. Death rates of children under the age of five vary across societies, in part due to differences in demographic (family's wealth and education) and regional characteristics [1]. For instance, the prevalence of child mortality is greater in rural regions of low- and middle-income countries because of numerous social factors, including early marriage, illiteracy, lack of access to maternal healthcare services (antenatal, birth and postnatal care) and malnutrition [2,3]. The Millennium Development Goals, long promoted by United Nations partners, aimed to decrease the number of child mortality by two-thirds from 1990 to 2015 [4]. Bangladesh was one of the few lower-middle-income countries that met all socio-economic MDG targets for 2015, including the reduction of child mortality. Specifically, child mortality in the country had decreased from 151 per 1000 live births in 1990 to 40 per 1000 live births in 2019 [5,6].

The long-established sociocultural and economic system in Bangladesh makes it difficult for Bangladeshi women to obtain independence. For example, early marriages and dowry payments are widespread in certain areas of South Asian countries including Bangladesh, which often exposes women to abusive relationships. Domestic violence is a civil rights violation, often resulting in poor health outcomes for the survivor, and can affect anyone irrespective of ethnicity, culture or religion [7]. Bangladesh is a gender-restricted country; as such, Bangladeshi women, particularly in rural and suburban areas, are mostly confined to their homes, enjoy limited freedom and exercise little to no influence on decision-making, resulting in their propensity to regard themselves as delicate, needy and restricted to the role of raising children and performing household chores [[8], [9], [10]].

Health and socioeconomic factors of infant mortality have received substantial research attention, but few investigations have sought to understand the influence of social environment on child mortality. One of the societal issues affecting women's well-being is sexual abuse. Studies have estimated that 15%–71% of females are physically and/or sexually abused [4,11]. Women that are victimized by violence suffer acute trauma and long-term consequences, including a greater risk of further physical assault and mental health issues that could affect their children. Previous research has shown that a mother’s exposure to abuse harms her children's development [12,13]. The likelihood of someone being maltreated or abused increases with the presence of past exposure to violence as well as the magnitude of the violence [14]. More specifically, a child's psychological well-being is linked to their parents' violent tendencies [15]. Equally, the well-being of a mother is a critical determinant of her children’s health [16]. Given the link between family violence and the health of newborns and children as well as child mortality, our research aims to examine the impact of social factors including women’s experiences and views of family violence on child mortality.

### 1.1 Prior research

Children's mortality is influenced by a variety of factors that are closely linked to demographic variables, such as the mother's age at birth, the gender of the child and number of children ever born. Mothers who give birth to children when they are very young are at increased risk of death, although the mortality rate is higher for older women (greater than 40 years) than for younger mothers [17]. Child and forced marriage is a significant human rights violation and public health issue worldwide. It has negative effects on the women, such as unplanned and high-risk pregnancies. The risk of IPV, which has been related to negative physical and mental health consequences, is higher in early marriages, particularly for brides under the age of 15 years [18,19]. Many studies have also shown that newborn and child mortality is highest among first-born children, but comparatively lower among second and third children [20]. Child mortality is negatively associated with the duration of the birth interval (i.e., the shorter the gap between births, the greater the risk of death for children under the age of 5 years) [21]. Salmon [22], discovered the length of birth determines individual personality characteristics. Birth interval significantly influences women’s mental health and lifestyle choices, which in turn affects how they cope with life's events [23].

Female literacy has a crucial influence on the general growth and development of society. Extensive studies have shown that children who are cared for by literate mothers have complete, balanced development in all elements of their lives. Infant and maternal mortality rates continue to decrease, partly due to rising female literacy, which improves health indicators for both mothers and children [24]. Higher levels of parental education have been shown to have a significant and persistent influence on child survival [25]. Educated women utilize knowledge more successfully while caring for their children and seek appropriate health treatment more effectively than uneducated mothers [26]. Additionally, Intimate Partner Violence (IPV) is associated with women's literacy, although there are additional complexities to this link. A review of available literature at the individual level indicates that women's educational attainment may have a protective impact against IPV [27,28].

In the context of South Asian countries, studies have found child mortality and IPV to be influenced by regions, with rural areas reporting greater rates of child mortality and IPV compared to urban areas [[29], [30], [31]]. The rigid social norms that condone violence in families change more slowly in rural regions than they do in urban ones, resulting in rural women being more vulnerable to IPV than their counterparts in urban areas [32]. Under-five mortality rates in rural areas were higher for children of mothers who are younger and lack or have limited access to proper sanitation, electricity and health facilities. Meanwhile, in urban regions, children born to working mothers married to educated husbands and those born in upper-income households had reduced probabilities of dying before the age of 5 [33]. The poor economic status of women can also contribute to child mortality. Due to their precarious financial circumstances, Bangladeshi women have historically viewed themselves as dutiful wives who raise their children and take care of household responsibilities [9] and are often subjected to widespread violence in all aspects of their life. Meanwhile, studies have shown that a child’s health is at risk when their mother experiences any type of family or social violence [34].

Although some studies have identified demographic and socioeconomic determinants of child mortality in Bangladesh, we believe that many health-related and societal aspects have changed as a result of the continuous interventions by government and Non-Governmental Organizations (NGOs) over the past few decades. Therefore, it is worthwhile to continue to investigate child mortality, especially in relation to women’s exposure to family violence, as few studies to date have assessed the connection between child mortality and women’s perceptions of family violence in Bangladesh. Having said that, the purpose of this research is to evaluate how women’s attitudes toward partner violence and their socioeconomic and demographic characteristics contribute to the death of children under the age of 5 years in Bangladesh. The results of this study will assist policymakers in taking the required steps to accelerate the reduction in mortality and IPV rates across the country.

## 2 Methods

### 2.1 Sampling design and variables

The present study employed a nationally representative survey dataset from the MICS 2019, which provides a wide range of information, including factors related to child mortality (e.g., the mother’s age at birth and age at first marriage) and socioeconomic characteristics (e.g., household income, mother’s education and mothers’ perceptions of IPV). The survey included a total of 3220 primary sampling units and 64,400 sampled homes. The urban and rural regions within each district were chosen as the primary sampling stratum, and a sample of households was selected in two stages: first, in the urban areas and then in the rural areas. Within each stratum, a predetermined number of census enumeration areas were chosen in a methodical manner with a probability proportionate to the size of the stratum in which they were located. Following the completion of a household listing within the chosen enumeration regions, a systematic sample of 20 homes was taken from each of the sample PSUs in a random manner. Because the sample is not self-weighting, sample weights were employed in the present study.

**Fig. 1:**
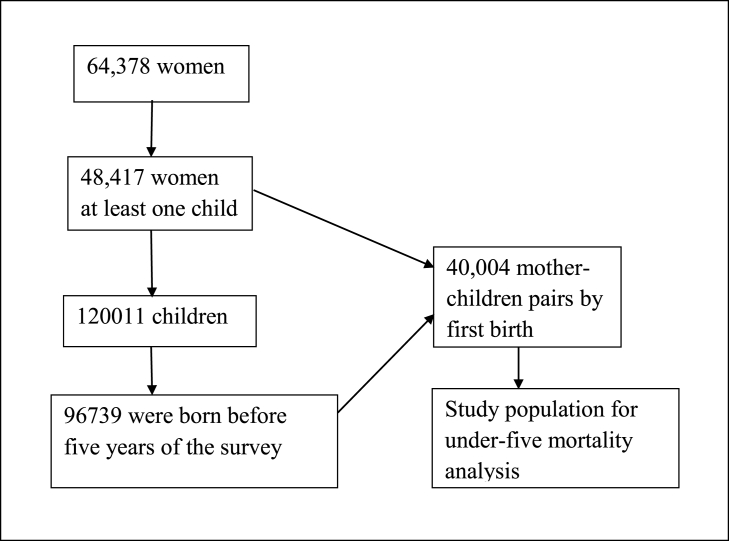
Flow chart for the selection of the study participants, MICS-2019.

The MICS 2019 used five questionnaires to collect information. This study used the women and children’s datasets and merged them into one. A total of 64,378 women participated in the survey, of which 48,417 women satisfied the condition of birthing at least one child. The MICS collected information on 120,011 children, of which 96,739 were born five years before the survey. Among the 96,739 children, 6225 died before reaching the age of 5 years, while 90,514 were alive at the date of the survey. However, for the analysis of child mortality, we utilized data of only the firstborn children, which resulted in 40,004 combined datasets of mothers and children (Fig. 1) [35]. Data of mothers and firstborn children were considered because mortality risks are high among first births of predominantly younger mothers [20,36,37].

The survey protocol was approved by the technical committee of the Government of Bangladesh led by the Bangladesh Bureau of Statistics. The protocol included a Protection Protocol detailing possible hazards in the survey life cycle and management strategies to mitigate these threats. To ensure confidentiality, verbal permission was obtained from each respondent and parental approval was obtained for all child participants. All data were fully anonymized by the MICS authority prior to making them available.

### 2.2 Outcome variable

The outcome variable of this study is the mortality rate of children below 5 years of age. Mortality was dichotomized into two responses: ‘1’ if the child had died before reaching the age of 5 and ‘0’ otherwise.

### 2.3 Predictor variables

In this analysis, the following socioeconomic and demographic variables for women were considered as regressors: education level, age at first marriage, internet usage, place of residence (urban vs. rural), division (first-level administrative area), income status, age at first birth (having a child at a young age is associated with unfavorable outcomes for the baby's health), gender of the child (first-born), number of children ever born, and access to mass media. Most of the selected variables were categorical, and the others were classified as continuous variables for the analysis.

### 2.4 Statistical analysis

We divided our analysis into two parts: women’s attitudes toward domestic violence and vulnerable women who had been physically attacked. We considered the vulnerability of women to investigate if the determinants of child mortality related to the attitudes of women toward IPV were reflected in the number of women who had been physically attacked. Women’s attitudes toward domestic violence were obtained from the following five questions of the MICS dataset [ [6,38,39]].

1. If she goes out without telling husband: wife beating justified.
2. If she neglects the children: wife beating justified.
3. If she argues with husband: wife beating justified.
4. If she refuses sex with husband: wife beating justified.
5. If she burns the food: wife beating justified.

Responses were coded ‘1’ if the answer was “yes” and ‘0’ if otherwise. The answers were then summed and coded as “not justified’ if the sum was 0, “justified” if the sum was 5, and “somewhat justified” if the sum is between 1 and 4.

Women were also asked if they had been physically attacked three years before the survey either inside or outside of their homes. The study considered attack incidents (excluding robbery) in which the respondent was the victim. Responses were coded as “yes” or “no.”

Data regarding child mortality were expressed as frequencies for the number of deaths of children under 5 years and the number of surviving children. In this instance, the count regression model performs much better in terms of analyzing the response data. We used a Poisson regression model to identify the socioeconomic and demographic factors responsible for child mortality. A Poisson regression model is one of the appropriate methods for analyzing binary responses for which the probability of success is very small [40,41]. In this analysis, the probability of child mortality in Bangladesh was comparatively lower than the probability of a child surviving up to 5 years. Moreover, a Poisson regression model has the strict assumption that the mean and the variance of response variables are equal. The mean and variance binary responses in this study were found to be almost equal (Table 2).

The probability function for Y is given as follows:

(1) $$\text{P}\left(\text{Y }=\mathrm{yi /\mu i}\right)\;=\;\frac{\exp\left(-\mathrm{\mu i}\right)\;{\mathrm{\mu i}}^{{\text{y}}_{\text{i}}}}{{\text{y}}_{\text{i}}!}$$

where i = 0,1,2,3,4 ….. and μi > 0, yi = 0,1,2,3 …..

Where, y_i_ in equation (1) is the number of children who died before the age of 5 years of the i-th mother in a given time with a mean parameter μ_i_. Notably, the mean and variance of the Poisson distribution are equal.

## 3 Results

### 3.1 Demographic characteristics of women

The sociodemographic characteristics of participants are described in Table 1. The study found that above one-third (39.6%) of the women had secondary education, followed by primary education (28.8%) and tertiary education (8.6%). However, 23.0% of the women were uneducated.

**Table 1:** Sociodemographic Characteristics of Bangladeshi Women (Aged 15–49 Years) and their Attitude and Exposure to IPV, 2019.

Variables	Number (percentage) of women	Percentage distribution of women’s attitude toward partner violence (hitting or beating by husbands)			Percentage of vulnerable women who had been physically attacked	
		Not justified	Somewhat Justified	Justified	Yes	No
**Education**						
No Education	9197(23.0)	62.3	30.1	7.6	4.8	95.2
Primary	11529(28.8)	66.5	28.8	4.7	5.9	94.1
Secondary	15851(39.6)	74.1	23.5	2.5	4.3	95.7
Higher Secondary+	3427(8.6)	86.8	11.8	1.4	2.0	98.0
**Age at first marriage (in years)**						
<18	27571(68.9)	68.9	26.9	4.2	5.3	94.7
18+	12433(31.1)	73.3	22.5	4.2	3.3	96.7
**Internet use**						
No	36167(90.4)	69.1	26.4	4.5	4.9	95.1
Yes	3838(9.6)	81.0	17.4	1.6	2.3	97.7
**Place of residence**						
Urban	8945(22.4)	74.1	22.6	3.3	3.2	96.8
Rural	31060(77.6)	69.2	26.4	4.4	5.1	94.9
**Division**						
Barishal	2208(5.5)	69.5	28.8	1.7	3.1	96.9
Chattogram	7364(18.4)	74.2	22.8	3.0	2.0	98.0
Dhaka	9928(24.8)	70.3	25.9	3.8	4.1	95.9
Khulna	5078(12.7)	75.6	23.1	1.3	7.6	92.4
Mymenshing	2584(6.5)	75.7	20.0	4.3	2.6	97.4
Rajshahi	5654(14.1)	62.3	35.5	2.1	5.5	94.5
Rangpur	4687(11.7)	60.7	27.6	11.7	9.7	90.3
Sylhet	2501(6.3)	78.7	13.4	7.9	1.4	98.6
**Wealth index**						
Poor	15682(39.2)	63.4	30.7	5.8	6.8	93.2
Middle	16125(40.3)	71.4	25.1	3.5	4.2	95.8
Upper	8197(20.5)	81.2	16.5	2.3	1.6	98.4
**Mother’s age at first birth (in years)**						
<20	25377(63.4)	68.9	26.9	4.2	5.2	94.8
20+	14628(36.6)	72.6	23.2	4.2	3.8	96.2
**Gender of child**						
Boy	20507(51.3)	70.1	25.5	4.4	4.7	95.3
Girl	19498(48.7)	70.4	25.6	3.9	4.7	95.3
**Children ever born**						
1-2 child	20342(50.8)	71.9	24.7	3.3	4.8	95.2
3-6 child	19040(47.6)	68.6	26.5	4.9	4.6	95.4
7+	622(1.6)	67.4	23.6	9.0	4.0	96.0
**Access to mass media**						
No access at all	13136(32.8)	64.7	29.8	5.5	5.1	94.9
Limited access	24868(62.2)	71.7	24.5	3.8	4.7	95.3
Full access	2000(5.0)	88.6	10.2	1.2	2.0	98.0
**Women’s attitude toward domestic violence**						
Not justified	28110(70.3)	–	–	–	4.3	95.7
Somewhat Justified	10219(25.5)	–	–	–	5.5	94.5
Justified	1675(4.2)	–	–	–	5.5	94.5
**Women who had been physically attacked**						
Yes	1874(4.7)	65.0	30.1	4.9	–	–
No	38131(95.3)	70.5	25.3	4.2	–	–
Total Woman	40004	28110	10219	1675	1874	38131

Source: Author’s elaboration using MICS 2019 survey dataset.

**Table 2:** Percentage distribution of under-five child death in Bangladesh, 2019.

Variables	*Percentage of under-five death	Percentage of survival at least 5 years	Variables	*Percentage of under-five death	Percentage of survival at least 5 years
Mother’s education			Gender of child		
No Education	11.7	88.3	Boy	8.5	91.5
Primary	8.1	91.9	Girl	6.2	93.8
Secondary	5.3	94.7	**Women’s attitude towards domestic violence**		
Higher Secondary+	2.6	97.4	Not justified	7.1	92.8
**Mother’s age at first marriage (in years)**			Somewhat Justified	7.7	92.3
<18	8.2	91.8	Justified	9.4	90.6
18+	5.5	94.5	**Women who had been physically attacked**		
**Mother’s internet use**			Yes	9.3	90.7
No	7.8	92.2	No	7.3	92.7
Yes	3.7	96.3	**Mother's age at first birth (in years)**		
**Place of residence**			<20	8.5	91.5
Urban	5.5	94.5	20–34	5.3	94.7
Rural	7.9	92.1	**Wealth Index**		
**Division**			Poor	9.3	90.7
Barishal	6.4	93.6	Middle	7.2	92.8
Chattogram	7.0	93.0	Upper	4.1	95.9
Dhaka	6.5	93.5	**Children ever born**		
Khulna	7.1	92.9	1-2 child	1.9	98.1
Mymenshing	9.4	90.6	3-6 child	12.6	87.4
Rajshahi	7.6	92.4	7+	28.3	71.7
Rangpur	8.1	91.9	Total	2946(7.4%)	37058(92.6%)
Sylhet	9.4	90.6			
**Access to mass media**					
No access at all	8.8	91.2			
Limited access	6.9	93.1			
Full access	3.2	96.8			
**Response variable, Under 5 child mortality ‘1 = yes’ and ‘0 = No’** **N = 40004, Mean = 0.07 and Variance = 0.068**					

Source: Author’s elaboration using MICS 2019 survey dataset.

The majority of the women were married before reaching the legal marriage age of 18 years (68.9%), lived in rural areas (77.6%) and had never used the internet (90.4%). In this study, 24.8% of the women were from the Dhaka division, while 18.4% and 14.1% of the women were from Chattogram and Rajshahi, respectively. The results of respondents’ economic conditions showed that 40.3% were from middle-income households, 39.2% were from low-income households and 20.5% were from upper-income households. Among the women, 63.4% had begun childbearing before reaching the age of 20 years, 51.3% of their first-born children were male and 47.6% had birthed between three and six children. Responses concerning the socioeconomic variable of access to mass media indicated that 32.8% of the women had no access to any type of mass media, such as television, print media or radio. Above two-third (70.3%) of the women had negative perception towards IPV, whereas approximately one-fourth (26%) considered justification in certain circumstances. On the other hand, 95.3% of the women were not attacked physically three years before the survey.

### 3.2 Women’s attitudes toward domestic violence

Table 1 also presents the percentage distribution of women’s attitudes toward domestic violence in relation to their socioeconomic and demographic conditions. The results showed that among uneducated women, 62.3% believed IPV is not justified in any of the five provided scenarios (i.e., a sum of ‘0’ for their responses), while 7.6% reported that IPV is justified in all the cases (i.e., a sum of ‘5’ for their responses). On the other hand, among women with primary education, 66.5% declared that IPV was unjustified, whereas 4.7% justified IPV. This indicates that the percentage of women justifying IPV decreases with increasing education levels. At the highest level of education, only 1.4% of women considered IPV to be justified, as opposed to 86.8% who considered it to be unjustified. Of the women who married after the legal age of marriage in Bangladesh, 73.3% disapprove of IPV, while 22.5% considered IPV to be somewhat justified.

Access to internet use and the wealth index appear to be important factors when considering the perceptions of women toward IPV. Table 1 shows that more than 80% of respondents who have used the internet believed IPV was never justified. The same result (more than 80%) was found among women from upper-income households. On the other hand, the proportion of rural women disproving IPV was 69.2%. The divisional area data indicated that the highest proportion of women who strongly believed IPV is justified reside in the Rangpur division (11.7%), followed by Sylhet (7.9%), whereas the lowest proportion was found among women living in the Khulna division (1.3%). However, the highest proportion of women who hold that IPV is somewhat justified was from the Rajshahi division (35.5%), followed by Barisal (28.8%) and Rangpur (27.6%).

Table 1 also indicates that among different age groups of mothers at the time of the birth of their first child, two-thirds maintained that IPV is unjustified, whereas one-third considered IPV to be somewhat justified. Among mothers whose firstborn child was male, 70.1% hold IPV to be unjustified, whereas 4.4% believed it is justified.

The variables of children and mothers’ health were considered in relation to the number of children ever born. Among women who had seven or more children, 9.0% of them reported that IPV is justified, whereas 3.3% of women who had two children or fewer responded the same. On the other hand, 88.6% of women who had frequent access to mass media were in full oppose of IPV, just like 64.7% of women who never had access to any media. Among women who had been physically attacked, 65.0% considered IPV to be unjustified, while 4.9% believed to be justified.

### 3.3 Physical violence (social vulnerability)

The percentage of women who had been physically attacked either inside or outside their homes is shown in Table 1. The data indicated that highly-educated women were less likely to be attacked than women with a lower level of education. Only 2.0% of women with higher education reported being physically attacked, whereas 5.9% of women with primary education and 4.8% of women with no education reported being attacked. Of women who married before the legal age of 18 years, 5.3% reported being attacked compared to 3.3% who married after the legal age. Women can protect themselves against all forms of violence if they have knowledge of new technology and how to obtain information from the internet. The study found that 4.9% of women who have never used the internet were physically attacked, whereas only 2.3% of women who have used the internet reported being attacked. In terms of geographical location, rural women (5.1%) recorded more physical attacks than their urban counterparts (3.2%). On the other hand, divisional variations indicated that the highest physical violence against women occurred in Rangpur (9.7%), followed by Khulna (7.6%), Rajshahi (5.5%), and Dhaka (4.1%). Moreover, women reporting household incomes in the upper quartile (1.6%) of the wealth index reported being attacked less often than women from households in the lower quartile (6.8%).

The study found that, of the women who were below the age of 20 years at the first birth of their child, 5.2% reported being physically attacked, compared to 3.8% of women whose age at first child birth was 20 years and above. Among the women who had seven or more children, 4.0% of them reported being physically attacked, similar to 4.8% of women who had two or fewer children. It was also found that 2.0% of women who had access to media reported being physically attacked, as opposed to 5.1% of women who had no access to any mass media. The analysis of women’s perceptions toward domestic violence indicated that, among women who believed IPV is justified, 5.5% had been physically attacked; whereas, among women who believed IPV is never justified, the incidence of physical attack was lower (4.3%).

### 3.4 Child mortality

Table 2 reports the child mortality rate in Bangladesh according to the MICS 2019. The study found that under-five mortality is highest (11.7%) among mothers who had no education and the lowest (2.6%) among mothers with at least higher secondary education. Among the mothers who married before the legal age in Bangladesh, 8.2% had experienced the death of a child under the age of 5 years. Additionally, 7.8% of women who never had access to the internet also experienced the death of a young child. In terms of geographical region, 7.9% of women from rural areas experienced the death of a young child. Among the divisions, the highest rates of child mortality were found in Sylhet (9.4%) and Mymenshing (9.4%) divisions. On the other hand, of the women who had never accessed any mass media, 8.8% experienced the death of a young child; whereas, among those who had limited mass media access, 6.9% had experienced under-five mortality. Only 3.2% of women who had full access to mass media had reported under-five child mortality.

In terms of the child sex, 8.5% of women whose first child was male experienced child mortality, whereas the rate was 6.2% for women whose child was female. The highest child mortality rates were found among mothers who thought IPV is justified (9.4%), were socially vulnerable (i.e., physically attacked; 9.3%), whose age at first birth was below 20 years (8.5%) and belong to low-income households (9.3%). The interesting part of the study is that, of the women who have at least seven children at the time of the survey, 28.3% experienced the death of a young child.

### 3.5 Poisson regression estimates for child mortality analysis

The Relative Risk (RR) estimates for child mortality based on sociodemographic and economic factors of women in Bangladesh using the Poisson regression model are presented in Table 3. The results indicated women’s level of education as being the most significant determinant of child mortality. Women with higher secondary education (RR = 0.713) were less likely to experience the death of a young child than women with no education. Among women who believed IPV was unjustified, women with higher degrees and secondary education were 31.0% and 19%, respectively, less likely to face child mortality than women with no education. In addition, among women who had reported they had never been physically attacked, the risk of child mortality was 30.0% lower for highly-educated women than those who had no education.

**Table 3:** Relative risk estimates for analysis of under-five child death in Bangladesh using the Poisson regression model.

Variables	Overall	Mother’s attitude towards domestic violence (beating by husband’s)			Mother had been physically attacked	
		Not justified	Somewhat Justified	Justified	Yes	No
**Mother’s education**						
No Education(R)	1.00	1.00	1.00	1.00	1.00	1.00
Primary	0.90**	0.88**	0.97	0.80	1.19	0.88***
Secondary	0.84***	0.81***	0.95	0.71	1.70	0.83***
Higher Secondary+	0.713***	0.69***	0.73	0.64	1.14	0.70***
**Mother’s age at first marriage (in years)**						
<18 (R)	1.00	1.00	1.00	1.00	1.00	1.00
18+	0.97	1.02	0.88	0.91	0.83	0.98
**Mother’s internet use**						
No (R)	1.00	1.00	1.00	1.00	1.00	1.00
Yes	0.83**	0.83*	0.77	1.13	1.20	0.82***
**Place of residence**						
Urban (R)	1.00	1.00	1.00	1.00	1.00	1.00
Rural	1.035	0.99	1.08	2.08**	0.78	1.05
**Division**						
Barishal (R)	1.00	1.00	1.00	1.00	1.00	1.00
Chattogram	1.11	1.10	1.21	1.75	1.21	1.12
Dhaka	1.38***	1.27**	1.61***	2.35	1.65	1.37***
Khulna	1.55***	1.45***	1.78***	2.99	1.51	1.56***
Mymenshing	1.46***	1.32**	2.01***	1.79	1.00	1.47***
Rajshahi	1.52***	1.57***	1.47**	1.49	1.68	1.51***
Rangpur	1.38***	1.42***	1.20	2.31	1.67	1.36***
Sylhet	1.35***	1.27*	1.59**	2.29	1.04	1.35***
**Wealth index**						
Poor (R)	1.00	1.00	1.00	1.00	1.00	1.00
Middle	0.93*	0.93*	0.94	0.85	1.01	0.92*
Upper	0.73***	0.71***	0.83	0.63	0.55	0.74***
**Mother's age at first birth (in years)**						
<20 (R)	1.00	1.00	1.00	1.00	1.00	1.00
20+	0.78***	0.78***	0.81**	0.70	0.67	0.79***
**Gender of child**						
Boy(R)	1.00	1.00	1.00	1.00	1.00	1.00
Girl	0.68***	0.68***	0.69***	0.68**	0.94	0.67***
**Children ever born**						
1-2 child (R)	1.00	1.00	1.00	1.00	1.00	1.00
3-6 child	6.42***	6.14***	7.69***	4.91***	7.10***	6.38***
7+	13.30***	11.50***	17.52***	14.43***	22.48***	12.90***
**Access to mass media**						
No access at all	1.00	1.00	1.00	1.00	1.00	1.00
Limited access	0.80***	0.78***	0.95	0.55***	0.91	0.80***
Full access	0.39***	0.38***	0.55*	0.01	0.76	0.38***

Source: **Author’s elaboration using MICS 2019 survey dataset**.Note: R = Reference Category, *p < 0.10, **p < 0.05; ***p < 0.01.

Moreover, internet use can reduce the risk of child mortality by helping parents obtain instant health information. The study found that women who, apart from opposing IPV and had never been physically attacked, used the internet were less likely to experience child mortality than those who did not use the internet (RR: 0.82–0.83). Further, among women who considered IPV to be justified, those from rural areas were 2.08 times more likely to face child mortality than those in urban areas. Regional variation is another significant determinant of child mortality. Compared with the Barishal division in Bangladesh, women in all other divisions (except Chattogram) were more likely to face child mortality (RR: 1.35–1.55). The same scenario was found for women who believed IPV was unjustified and those who had never been physically attacked.

The socioeconomic determinant of the wealth index indicates a negative association between social status and child mortality. To illustrate, among women who believed IPV was unjustified, those from middle- and upper-income households were 7% and 29%, respectively, less likely to experience under-five mortality than those from lower-income households. Among women who had never been physically attacked, those from upper-income households were 26% less likely to experience child mortality than women in the reference group. On the other hand, of women who disapprove of IPV, those whose first birth was at the age of 20 years or older had a lower risk of child mortality (RR: 0.78) than those who birthed their first child before the age of 20 years. However, irrespective of the mother’s perceptions toward IPV or exposure to physical attack, female infants had a lower risk of child mortality (RR: 0.67–0.69) than male infants in Bangladesh. Furthermore, women with more than two children were more likely to experience child mortality (RR: 4.91–7.69 for 3–6 children; RR: 11.50–22.48 for at least seven children) than those who had two or fewer children despite their disproval of IPV or lack of exposure to physical attack. Lastly, women who had frequent access to mass media were less likely to experience child mortality than those who had no access to mass media regardless of their perception of IPV.

## 4 Discussion

This study examined child mortality and the influence of women’s perception of IPV. Our findings suggest that women’s attitudes toward IPV were a significant factor influencing child mortality, as a substantial number of children’s deaths were reported at the time of the survey from those women who had positive attitudes toward IPV. Noticeably, women who believed IPV was unjustified, of those possess higher education have less risk of child mortality than uneducated women. This finding indicates that education can minimize the adverse effect of maternal exposure to violence on child mortality [38,42]. Elimination of barriers to higher education for women might prevent childhood death as well as increase the safety of women and should be the primary focus of policy intervention. Women's education can be supported by providing them with financial incentives such as scholarships and stipends in higher education, which may contribute to their families' economic well-being [43]. The benefits of maternal education include increased wealth and literacy, lower fertility, delayed marriage and childbearing, greater health-related knowledge, improved health-seeking behaviors, and increased female empowerment. These benefits ultimately contribute to a reduction in the overall child mortality rate [44]. Therefore, the development of a safe environment for women’s education is crucial and requires the concerted efforts of different social institutions.

Our findings show that amid the risk of IPV, women who married before the legal marriage age of 18 years had a greater risk of child mortality compared to those married who married at 18 years or above. Women who marry before the legal age experience poorer health and have babies who are at greater risk for malnutrition [45]. The findings showed that violence toward women and access to the internet varies with geographical location, albeit their significant influence on child mortality [46,47]. Access to Information and Communication Technology (ICT) lowers health risks for children and ultimately reduces the rate of child mortality and also promotes the empowerment of women and the sustainability of their status in society. Urban women tend to be more cautious regarding their children’s health and social rights than rural women in Bangladesh [48]. Therefore, the establishment of an ICT center in rural areas and the minimization of existing disparities in the utilization of healthcare services between urban and rural regions should be prioritized. The availability of such facilities to all women will lower their susceptibility to IPV, which could ultimately decrease their child mortality risks.

Women in Dhaka, Khulna, Mymenshing, Rajshahi, Rangpur, and Sylhet divisions were more likely to experience child mortality than women in the Barishal division. A high rate of maternal believe towards justification of IPV as well as child death was found in the Rangpur and Sylhet divisions, which could be attributed to a variety of reasons, including religious influence, superstitions, and a lack of knowledge about child and maternal health care [49]. This suggests that to reduce under-five child mortality, regulations targeted at specific regions are required, with a particular emphasis on women who believed IPV is justified.

In Bangladesh, we found that the financial status of the family has a substantial impact on the women’s attitude towards IPV and also on the survival of their children. Children from higher-income families are more likely to survive than those from lower-income families [50]. The socioeconomic position of women, particularly their financial status, may be associated with good health practices, including child care and optimal eating habits. Moreover, the socioeconomic status of a woman may influence her role in the family and empower her to take steps to enhance the health of her children by ensuring that contemporary and innovative health services are used appropriately and timely [51,52].

The age of the mother at the time of birthing her first child was recognized as a significant explanatory variable. Mothers who were young (below age 20 years) at their first childbirth had a greater chance of experiencing child mortality than those who birthed their first child after the age of 20. Moreover, young mothers (age at first birth below 20 years) who believed IPV was unjustified and had not been physically attacked were more likely to experience child mortality than mothers whose age at first birth was 20 years and above [[53], [54], [55]]. The gender of the child was also a statistically significant predictor of childhood death. Compared to male children, female children were less likely to die during the first 5 years of life when mothers experienced physical violence [36]. One potential reason for the greater risk of male child fatalities is that females have a biological edge against numerous causes of death that males suffer. In addition, the study found that women who had birthed seven or more children had a higher risk of child mortality than those who had two or fewer children. This observation could be because a larger family size might increase gender discrimination in a family. Moreover, the higher number of children in a family seems to lower the family's quality of living, regardless of the country’s socioeconomic condition. Among women who had negative attitudes toward IPV, those who had frequent access to mass media had a lower chance of experiencing child mortality, as they can quickly obtain accurate health information concerning childhood diseases and other issues that affect the health of a child.

Significant mortality differentials were observed when considering the vulnerability of women, which was measured by women’s previous exposure to physical abuse. The findings revealed a higher risk of child mortality among women who had been physically attacked and believed IPV is justified. IPV has been associated directly (maltreatment and abuse of children) and indirectly (through incapacitation of women) with poorer health outcomes in young children [56,57].

This study has several strengths and limitations. The major strength of this study is that we used a nationally representative dataset with an appropriate method for describing child mortality that considered sample weights. This cross-sectional research uses a large random sample to generalize the population and is the first to investigate child mortality risk based on women’s perceptions toward IPV using the Poisson regression technique. The study's conclusions are legitimate for policymaking, since they were derived from an examination of large-scale data using an acceptable approach (the Poisson regression model). However, this research is based on childbirth data that was gathered 5 years before the survey, and the use of a cross-sectional dataset restricted our examination of other factors and establishment of causation or generalization to other populations. In addition, the survey collected the information from the respondents regarding physical attacked preceding three years of the survey which may create recall bias.

## 5 Conclusion

The result of this research offers significant policy implications, particularly in terms of identifying programs required for a sustained reduction in child mortality rates as well as the monitoring of public health. While concerned policies must consider personal and communal factors, it is crucial to go beyond these limits. Greater empowerment of mothers may assist in decreasing child mortality and also in promoting negative perceptions toward IPV, which ultimately enhance their status in the family and society. Moreover, better education and improved social status of mothers encourage their adoption of good health practices for themselves and their children, which could reduce the risk of child mortality in the long run.

According to the study, one-fourth of the women believed IPV was justified in certain circumstances, which is associated with the survival rates of their children. Our results suggest that women who had experienced physical abuse were more likely to experience child mortality. Moreover, women's education, use of the internet, social status, age at first birth, and access to mass media were significantly associated with their negative perceptions of IPV, which reduce child mortality. To decrease the chances of childhood death, stakeholders and policymakers should become involved in campaigns on criminalizing violence against women. More can be done to enhance the maternal status and child healthcare services by setting up information centers in rural areas, encouraging the use of mobile phones for different healthcare applications, reducing the cost of internet use, enforcing strict laws against marriage before the legal age, empowering women, reducing economic disparities across different divisions and discouraging larger family sizes by promoting family planning programs.

## Author contribution statement

Mohammad Nazmul Hoq: Conceived and designed the experiments; Analyzed and interpreted the data; Wrote the paper.

Ferdous Ara: Conceived and designed the experiments; Performed the experiments.

Mir Misnad Sultana: Performed the experiments.

Sabrina Naoshin: Analyzed and interpreted the data.

Israth Sultana: Contributed reagents, materials, analysis tools or data.

Mohammad Emdad Hossain: Analyzed and interpreted the data; Contributed reagents, materials, analysis tools or data.

## Funding statement

This research did not receive any specific grant from funding agencies in the public, commercial, or not-for-profit sectors.

## Data availability statement

This study extracted data from the secondary source of cross-sectional survey of Bangladesh Multiple Indicator Cluster Survey (MICS) 2019.

Data are available at https://mics.unicef.org/surveys.

The study was used the datasets upon the permission from the MICS authority.

## Declaration of interest’s statement

The authors declare no conflict of interest.
